# Supplementary material for: Content-rich biological network constructed by mining PubMed abstracts
Source: BMC Bioinformatics. 2004 Oct 8;5:147. doi: 10.1186/1471-2105-5-147 (PMC528731; doi:10.1186/1471-2105-5-147)
Supplement: Additional File 5 — The original Chilibot query results of the term "long-term potentiation (LTP)" and 22 other terms, limiting the latest references analyzed to the years 1990, 1995, 2000, and 2004. [file 1471-2105-5-147-S5.bz2 › chilibotAdditionalFile5/ltp1995/html/PI-3K_PKC.html]

 


 **PI-3K** and **PKC** 
  
Found 10 abstracts in PubMed,  **10 abstracts were retrieved and analyzed**.  


---

 Search Google  |
 PDF files only 
|  EDU domain only 

---

**Interactive relationship** (e.g. stimulation, inhibition, etc)

- These data indicate that PI3 kinase  [ **PI-3K** ]  activation is insensitive to  **PKC** .  Ref: 7933847 Kidney Int, 1994
- The isoquinoline sulfonyl piperazine compound H7 at concentrations that inhibit PDGF stimulated  **PKC**  activity had no effect on PDGF stimulated PI 3 kinase  [ **PI-3K** ]  activity in antiphospotyrosine immunoprecipitates.  Ref: 7933847 Kidney Int, 1994
- This kinase is not activated by diacylglycerol but is potently stimulated in vitro by the products of phosphatidylinositol 3 kinase PI 3 kinase  [ **PI-3K** ] , which suggests that zeta  **PKC**  is at least one of the critical targets of PI 3 kinase  [ **PI-3K** ]  triggered signals, and strengthens its role in cell proliferation.  Ref: 7989344 J Biol Chem, 1994
- The results suggest that wortmannin sensitive molecules such as PI3 kinase  [ **PI-3K** ] , are involved in the V1 receptor mediated activation of the MAP kinase pathway independent of TPA sensitive  **PKC** .  Ref: 8549762 FEBS Lett, 1995

- :-)

  **Parallel relationship** (e.g. studied together, co-existance, homology, etc.)

  - The EGF dependent GLUT4 translocation is possibly mediated by two independent pathways one by PI 3 kinase  [ **PI-3K** ]  and the other by protein kinase C  **PKC** .  Ref: 7999123 Biochem Biophys Res Commun, 1994
  - In conclusion, SCF induced PI 3 kinase  [ **PI-3K** ]  activation paralleled the increased SCF induced mitogenicity after inhibition of  **PKC** .  Ref: 7520444 J Biol Chem, 1994
  - These results suggest that wortmannin has a potent inhibitory effect on PI 3 kinase  [ **PI-3K** ]  and a weak inhibitory effect on  **PKC**  activity, and both effects cause a significant inhibition of insulin stimulated glucose uptake in rat adipocytes.  Ref: 8591706 Diabetes Res Clin Pract, 1995
  - We studied the effects of wortmannin on insulin and 12 O tetradecanoylphorbol 13 acetate TPA induced glucose uptake, purified  **PKC**  activity and in vitro 80 kDa protein phosphorylation to elucidate the relationship between insulin induced PI 3 kinase  [ **PI-3K** ]  and  **PKC**  activations.  Ref: 8591706 Diabetes Res Clin Pract, 1995
